# The Double-Edged Sword of SIRT3 in Cancer and Its Therapeutic Applications

**DOI:** 10.3389/fphar.2022.871560

**Published:** 2022-04-27

**Authors:** Shumin Ouyang, Qiyi Zhang, Linlin Lou, Kai Zhu, Zeyu Li, Peiqing Liu, Xiaolei Zhang

**Affiliations:** ^1^ National-Local Joint Engineering Laboratory of Druggability and New Drug Evaluation, Guangdong Key Laboratory of Chiral Molecule and Drug Discovery, School of Pharmaceutical Sciences, Sun Yat-Sen University, Guangzhou, China; ^2^ Innovation Practice Center, Changchun University of Chinese Medicine, Changchun, China

**Keywords:** SIRT3, mitochondria, deacetylase, activator, Inhibitor, cancer

## Abstract

Reprogramming of cellular energy metabolism is considered an emerging feature of cancer. Mitochondrial metabolism plays a crucial role in cancer cell proliferation, survival, and metastasis. As a major mitochondrial NAD^+^-dependent deacetylase, sirtuin3 (SIRT3) deacetylates and regulates the enzymes involved in regulating mitochondrial energy metabolism, including fatty acid oxidation, the Krebs cycle, and the respiratory chain to maintain metabolic homeostasis. In this article, we review the multiple roles of SIRT3 in various cancers, and systematically summarize the recent advances in the discovery of its activators and inhibitors. The roles of SIRT3 vary in different cancers and have cell- and tumor-type specificity. SIRT3 plays a unique function by mediating interactions between mitochondria and intracellular signaling. The critical functions of SIRT3 have renewed interest in the development of small molecule modulators that regulate its activity. Delineation of the underlying mechanism of SIRT3 as a critical regulator of cell metabolism and further characterization of the mitochondrial substrates of SIRT3 will deepen our understanding of the role of SIRT3 in tumorigenesis and progression and may provide novel therapeutic strategies for cancer targeting SIRT3.

## 1 Introduction

Belonging to an NAD^+^-dependent enzyme family, sirtuins (SIRTs) have various physiological functions similar to yeast Sir2. SIRTs possess various deacylase activities including deacetylase, desuccinylase, demalonylase, deureristoylase, demyristoylase, and depalmitoylase activities while SIRT4 and SIRT6 also have mono-ADP ribosyl transferase activity (North and Verdin, 2004; Yamamoto et al., 2007; Du et al., 2011; Jiang et al., 2013; Rack et al., 2015). SIRTs mediate cell proliferation, differentiation, apoptosis, inflammation, DNA repair, metabolism, and stress response, as well as carcinogenesis (O'Callaghan and Vassilopoulos, 2017; Preyat and Leo, 2013; Zhu et al., 2019). There are seven subtypes of SIRTs (SIRT1-SIRT7), with conserved functions and structures in mammals. The core domain of each protein comprises two subunits. The large structural domain is mainly composed of conservative Rossmann folds, while the small domain consists of a spiral structure and a zinc finger structure, which is less conserved. Crucially, a gap is formed between these two domains for substrate binding and catalysis (Hoff et al., 2006). This highly conservative core makes it difficult to design subtype-specific activators or inhibitors. The identification of the crystal structure of sirtuin3 (SIRT3) makes structure-based compound design feasible. Mammalian SIRTs have different tissue specificities, subcellular localizations, activities, and functions. SIRT1, SIRT6, and SIRT7 are mainly found in the nucleus, and SIRT2 is present in the cytoplasm. SIRT3, SIRT4, and SIRT5 are predominantly localized to mitochondria (Michishita et al., 2005). However, recent studies have shown that SIRT1 localization depends on the cellular context (Jin et al., 2007; Mattagajasingh et al., 2007; Tanno et al., 2007; Byles et al., 2010). SIRT1 is mainly located in the nucleus of normal cells but is found in the cytoplasm of cancerous cells (Byles et al., 2010). SIRT3 is mainly located in mitochondria, however, its presence in the nucleus and cytoplasm has also reported (Scher et al., 2007; Sundaresan et al., 2008; Iwahara et al., 2012) (Table 1).

**TABLE 1:** The characteristic of mammalian SIRTs.

Species	Localization	Activity	Function
SIRT1	Nucleus, Cytoplasm	Deacetylase	DNA repair, Metabolism, Inflammation, Apoptosis, Stress response, Adipogenesis, Genome stability
SIRT2	Cytoplasm	Deacetylase, Demyristoylase	Cell cycle, Carcinogenesis, Gluconeogenesis
SIRT3	Mitochondria, Nucleus, Cytoplasm	Deacetylase, Demyristoylase, Depaimitoylase	Metabolism, DNA repair, Neuroprotection, Oxidative stress, Tumorigenesis
SIRT4	Mitochondria	ADP-ribosyl transferase, Deacetylase, Lipoamidase	Insulin secretion, Metabolism, Tumorigenesis
SIRT5	Mitochondria	Demalonylase, Desuccinylase, Deacetylase, Deglutarylase	Ammonia detoxification, Metabolism
SIRT6	Nucleus	Demyristoylase, Depaimitoylase, ADP-ribosyl transferase, Deacetylase	DNA repair, Metabolism, TNF secretion, Tumorigenesis
SIRT7	Nucleus	Deacetylase	rRNA transcription, Carcinogenesis

**FIGURE 1:**
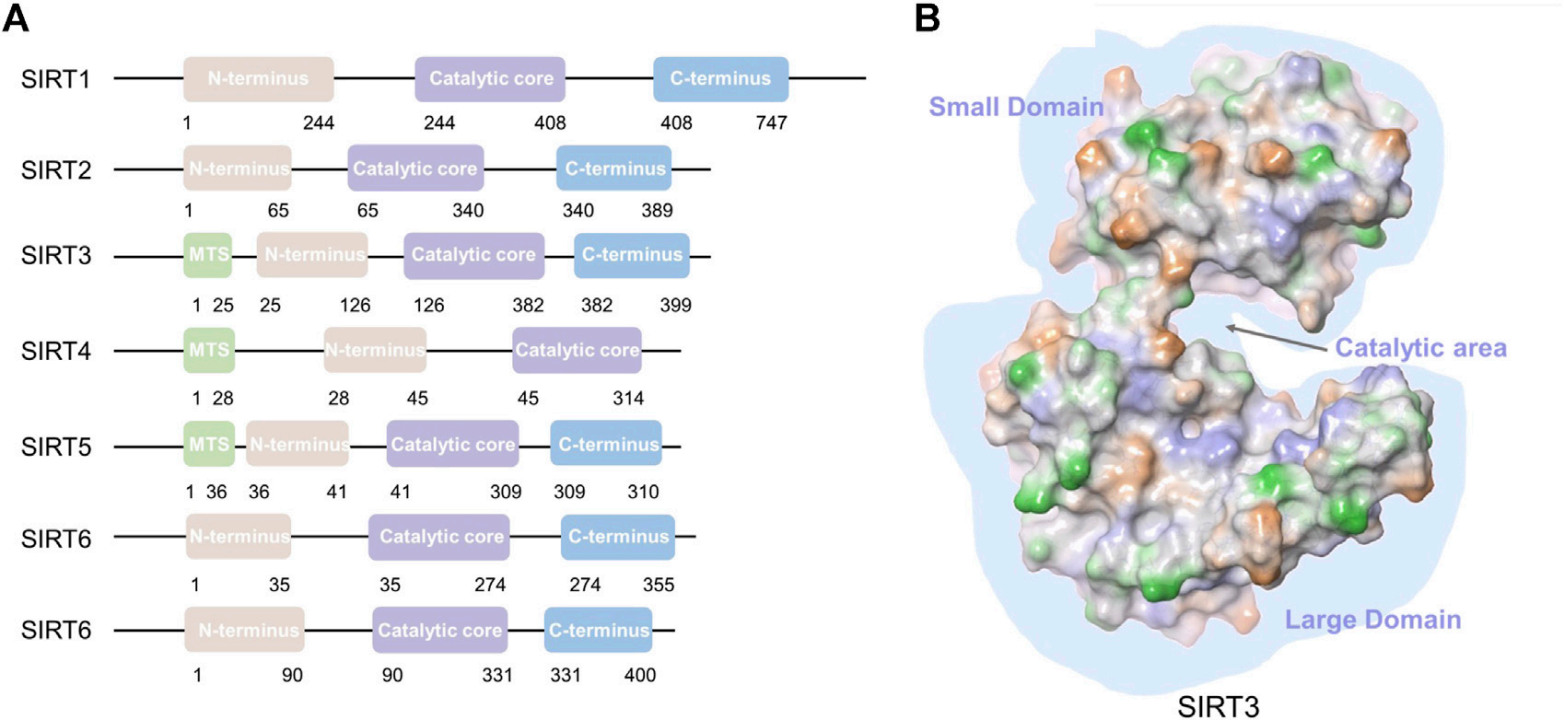
SIRT1-7 structures and SIRT3 domains. **(A)** SIRT1-7 structure; mitochondrial targeting sequence (MTS). **(B)** Major domains of the SIRT3 protein (PDB: 3GLS). A gap is formed between the small domain and large domain for substrate binding and catalysis.

In recent years, SIRT3-related research has intensified since its link to a longer lifespan in humans and its critical localization to the mitochondrial compartment have been recognized (Hurst et al., 2002). Age-associated pathologies and longevity are related to SIRT3 in humans. SIRT3 is the only member with strong NAD^+^-dependent deacetylase activity among the mitochondrial SIRTs (Lombard et al., 2007). SIRT3 consists 399 amino acids (Yang et al., 2010), including an N-terminal domain with a 25-amino-acid mitochondrial targeting sequence (MTS) (Onyango et al., 2002), a catalytic region, and a C-terminal domain (Figure 1). SIRT3 translocates to mitochondria with the N-terminal MTS and is cleaved into a 28 kD protein by mitochondrial processing peptidase (Schwer et al., 2002; Iwahara et al., 2012) to induce deacetylase functions (Smith et al., 2008). SIRT3 deacetylates various proteins that not only regulate mtDNA replication, transcription, translation, fatty acid oxidation, and amino acid metabolism, but also modulate enzymes that participate in the tricarboxylic acid (TCA) cycle, and regulate the activity of the electron transport chain (Kumar and Lombard, 2015; Lee, 2019). SIRT3 also plays a unique regulatory role by mediating interactions between mitochondria and intracellular signaling. SIRT3 is involved in the pathogenesis of diverse disorders including cancer, diabetes, neurodegeneration, cardiac hypertrophy, and liver steatosis. Surprisingly, SIRT3 acts as a double-edged sword in cancer. Thus, the development of compounds that alter SIRT3 activity has gained increasing attention from scientists. We will comprehensively review the mitochondrial substrates and functions of SIRT3, highlighting its dual role in various cancers, and summarize the activators and inhibitors of SIRT3 in this article.

## 2 The Main Targets and Substrates of Sirtuin3

SIRT3 is closely involved in the metabolism of amino acids, glycosides, and lipids in mitochondria (Hiromasa et al., 2004; Lombard et al., 2007). As an emerging, pivotal regulator of oxidative stress, SIRT3 regulates reactive oxygen species (ROS) production *via* deacetylation of key enzymes (Bause and Haigis, 2013). SIRT3 mediates the activities of isocitrate dehydrogenase (IDH) and manganese superoxide dismutase (MnSOD/SOD2) which are two major oxidative stress-responsive proteins, and SIRT3 is recognized as an important ROS scavenger in cells. Recent studies revealed that SIRT3 may regulate antioxidant-related enzymes by deacetylating and activating forkhead transcription factor (FOXO3a) (Sundaresan et al., 2009; Bause and Haigis, 2013; Tseng et al., 2013; Rangarajan et al., 2015). SIRT3 directly deacetylates and activates the enzymes involved in processes regulating mitochondrial energy metabolism, including fatty acid oxidation, the Krebs cycle, and the respiratory chain (Huang et al., 2010; Verdin et al., 2010; Torrens-Mas et al., 2017). The pyruvate dehydrogenase complex (PDC) is deacetylated by SIRT3, which funnels pyruvate to participate in the Krebs cycle, and protein kinase B is activated so that glucose uptake in glycolysis is accelerated (Hiromasa et al., 2004; Xu et al., 2019a). SIRT3 also deacetylates and activates long-chain acyl-CoA dehydrogenase (LCAD) (Hirschey et al., 2010), acetyl-CoA synthetase 2(AceCS2) (Schwer et al., 2006; Hirschey et al., 2010), and 3-hydroxy-3-methylglytaryl-CoA synthetase (HMGCS2) (Hirschey et al., 2010; Shimazu et al., 2010) to regulate lipid metabolism and fatty acid oxidation (Hirschey et al., 2010). Moreover, amino acid metabolism can be mediated by SIRT3 through deacetylation of glutamate dehydrogenase (GDH) (Lombard et al., 2007). SIRT3 deacetylates ornithine transcarbamylase (OTC) to regulate the urea cycle (Hallows et al., 2011).

**FIGURE 2:**
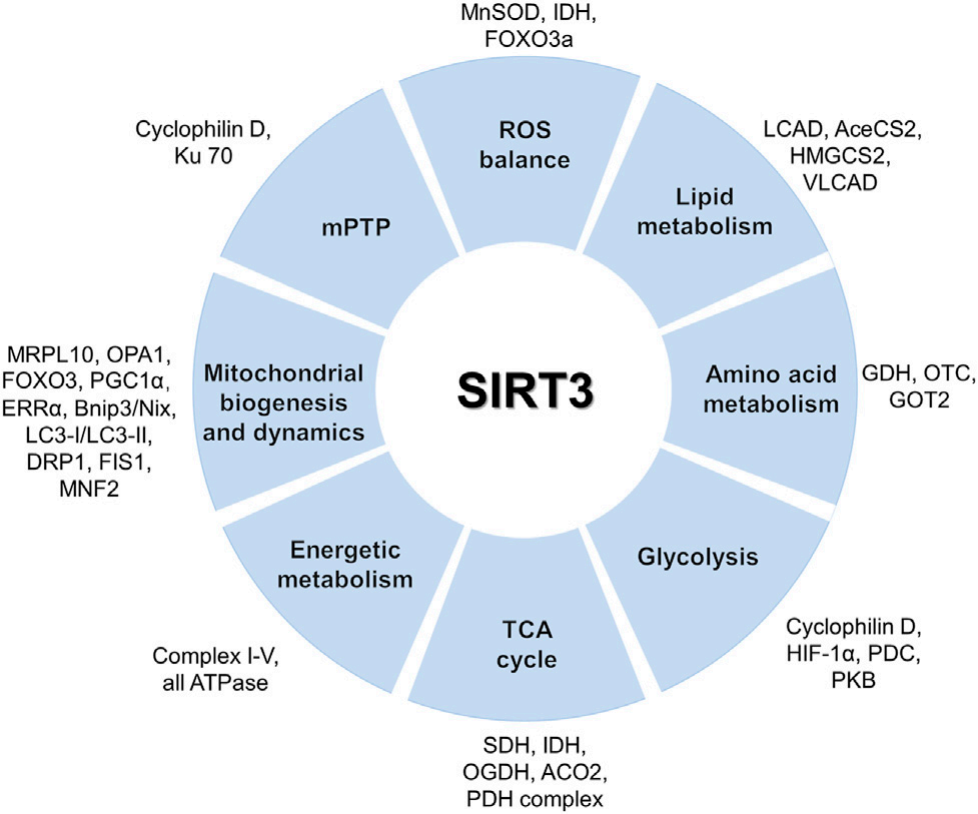
Mitochondrial functions and targets of SIRT3. SIRT3 activates various substrates *via* deacetylation to maintain metabolic homeostasis including substrates involved in the TCA cycle, glycolysis, amino acid metabolism, lipid metabolism, ROS balance, and mitochondrial dynamics.

Furthermore, SIRT3 promotes the progression of the TCA cycle through deacetylation of succinate dehydrogenase (SDH (Cimen et al., 2010)) and IDH (Meng et al., 2019). SIRT3 deacetylates numerous complex I–V subunits to regulate the oxidative respiratory chain (Ahn et al., 2008; Finley et al., 2011a) and ATPase (Wu et al., 2013). In addition, SIRT3 participates in mitochondrial biogenesis and dynamics (Torrens-Mas et al., 2017). SIRT3 also participates in the maintenance of mitochondrial quality. For instance, it was reported that SIRT3 coordinates the mitochondrial unfolded protein response (mtUPR) and upregulates mitochondrial autophagy (Papa and Germain, 2014). The mtUPR promotes a complex transcription program ultimately increasing mitochondrial integrity and fitness in response to oxidative proteotoxic stress. The activation of the SIRT3 axis of the unfolded protein response is linked to metastasis (Kenny et al., 2017). SIRT3 is the key coordinator of the mtUPR induced by mitochondrial protein cytotoxic stress (O'Callaghan and Vassilopoulos, 2017; Xu et al., 2019b; Weng et al., 2020). Moreover, SIRT3 can prevent mitochondrial dysfunction through the regulation of the mitochondrial permeability transition pore (mPTP) (Hafner et al., 2010; Torrens-Mas et al., 2017). Taken together, through reversible acetylation of mitochondrial proteins, SIRT3 plays a key role in mitochondrial metabolism. The main mitochondrial targets of SIRT3 are shown in Figure 2.

## 3 The Bioactivity and Function of Sirtuin3

SIRT3 mediates numerous biological and pathophysiological activities through the regulation of various biological functions, including repair, energy homeostasis, oxidative stress tolerance, apoptosis, enhanced longevity, anti-inflammatory responses, and other mechanisms (Ansari et al., 2017).

As a major repair enzyme in the base excision repair pathway, 8-oxoguanine DNA glycosylase (OGG1) is a newly discovered target of SIRT3. Cheng et al. found that SIRT3 deacetylates OGG1 to regulate its incision activity and decrease its degradation. Mitochondrial DNA damage repair, mitochondrial integrity, and apoptotic cell death induced by oxidative stress are closely related to the acetylation- and turnover-related regulation of OGG1 by SIRT3 (Cheng et al., 2013). Furthermore, SIRT3 has been reported to protect diverse types of cells from programmed cell death initiated by genotoxicity or oxidative stress by regulating ROS production and the mPTP and preventing Bax translocation to mitochondria (Pellegrini et al., 2012). Another study showed that SIRT3 acts as an anti-apoptotic protein. It was reported that SIRT3 can protect cells from death when mitochondrial NAD^+^ levels are severely decreased, in response to DNA damage (Yang et al., 2007).

SIRT3 is one of the most significant regulators of metabolism (Ansari et al., 2017). Ahn et al. implemented SIRT3 knockout mice to reveal the roles of SIRT3 in physiology. They demonstrated that SIRT3 is crucial to the regulation of basal ATP levels and energy homeostasis (Ahn et al., 2008). SIRT3 can regulate different enzymes including manganese dismutase, SOD2, and catalase, which are important in regulating ROS levels (Merksamer et al., 2013). More about the role of SIRT3 in metabolism is described in Part 2.

Given the high levels of SIRT3 in long-lived individuals, scientists suspect that SIRT3 is potentially associated with longevity. Initially, the discovery of unique single nucleotide polymorphisms (SNPs) linked to centenarians aroused interest in SIRT3 and its role in the human life span (Hurst et al., 2002; Rose et al., 2003; Bellizzi et al., 2005). Males carrying the G477T transversion in exon 3 of SIRT3 live longer than average. However, this nucleotide transition does not change the amino acid sequence and therefore is a silent change (Rose et al., 2003). The second SNP involves the variable number of tandem repeats (VNTR) region within SIRT3 intron 5, and specific VNTR polymorphisms show increased SIRT3 expression and have been linked to increased longevity (Bellizzi et al., 2005). The third SNP, unlike the previous two SNPs, has been associated with an increased risk of age-related metabolic syndrome. Some SNPs induce amino acid substitutions in the conserved deacetylase region, thereby reducing SIRT3 deacetylase activity (Hirschey et al., 2011). Therefore, specific SNPs of SIRT3 are associated with increased human lifespan, but more research is required to determine the mechanisms by which various SNPs affect human lifespan (Kincaid and Bossy-Wetzel, 2013). In addition, it was reported that a product of the FOXO transcription factor family helps to regulate the longevity of nematodes (Shi et al., 2005). Among FOXO family members, FOXO3a is a key substrate for SIRT3. SIRT3 interacts with FOXO3a to activate antioxidant genes, such as MnSOD and catalase, the products of which can reduce the level of ROS and positively affect disorders such as interstitial fibrosis and cardiac hypertrophy (Sundaresan et al., 2009).

SIRT3 not only has NAD^+^ dependent deacetylase activity but also catalyzes the removal of long-chain fatty acyl groups. These include myristoyl, palmitoyl, and others (Feldman et al., 2013). Acylation of long-chain fatty acids can regulate biological factors, including membrane associations, protein-protein interactions, and subcellular location. Gai et al. demonstrated that SIRT3 is an effective demyristoylase and is an expeditious depalmitoylase. Furthermore, they analyzed the crystal structures of SIRT3 in compound material containing a palmitoylated peptide or myristoylated H3K9 (Gai et al., 2016). The palmitoyl and myristoyl groups bound to the C-pocket and an allosteric site, respectively (Gai et al., 2016). However, the effects of demyristoylation, depalmitoylation, and other modifications on the functions of SIRT3 remain unclear.

## 4 The Roles of Sirtuin3 in Cancer

**FIGURE 3:**
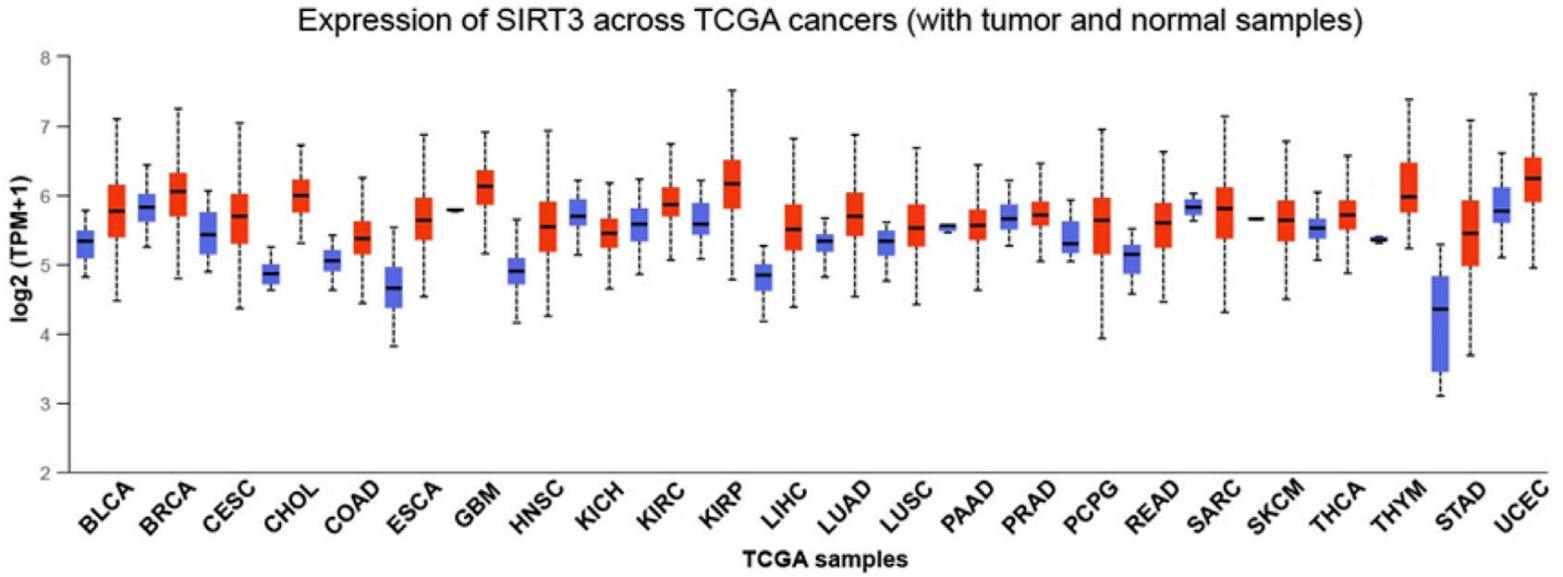
Expression of SIRT3 in different cancers. The expression of SIRT3 in different cancers with tumor and normal samples. Red indicates tumor samples and blue indicates normal samples. BLCA, bladder urothelial carcinoma; BRCA, breast invasive carcinoma; CESC, cervical squamous cell carcinoma; CHOL, cholangiocarcinoma; COAD, colon adenocarcinoma; ESCA, esophageal carcinoma; GBM, glioblastoma multiforme; HNSC, head and neck squamous cell carcinoma; KICH, kidney Chromophobe; KIRC, kidney renal clear cell carcinoma; KIRP, kidney renal papillary cell carcinoma; LIHC, live hepatocellular carcinoma; LUAD, lung adenocarcinoma; LUSC, lung squamous cell carcinoma; PAAD, pancreatic adenocarcinoma; PRAD, prostate adenocarcinoma; PCPG, pheochromocytoma, and paraganglioma; READ, rectum adenocarcinoma; SARC, sarcoma; SKCM, skin cutaneous melanoma; THCA, thyroid carcinoma; THYM, thymoma; STAD, stomach adenocarcinoma; UCEC, uterine corpus endometrial carcinoma.

Cancer is the most common cause of death worldwide. Carcinogenesis is characterized by cell enrichment, excessive proliferation, apoptosis resistance, and metabolic instability. Otto Warburg found that cancer cells have different metabolism patterns than normal tissues in the 1920s. Cancer cells consume many nutrients and rewire their metabolic processes to enable biosynthesis, which allows them to meet their energy and biomass production requirements (Finley and Haigis, 2012). Elevated ROS production, which is frequent in human malignancies, can promote tumorigenesis through a variety of mechanisms (Liou and Storz, 2010). ROS regulate cell proliferation, survival, differentiation, metabolism, and inflammation (Liou and Storz, 2010). Oxidative damage to DNA, lipids, and proteins can disrupt cellular processes, and further promote tumor progression (Finley and Haigis, 2012). Cancer is characterized by the reprogramming of cellular energy metabolism. SIRT3, regarded as a novel and potential therapeutic target, is shown to be involved in most of these cancer pathways. In addition, SIRT3 is abnormally expressed in a variety of cancers as shown in Figure 3. In this review, we emphasize the role of SIRT3 in tumorigenesis and cancer therapy.

### 4.1 Breast Cancer

**FIGURE 4:**
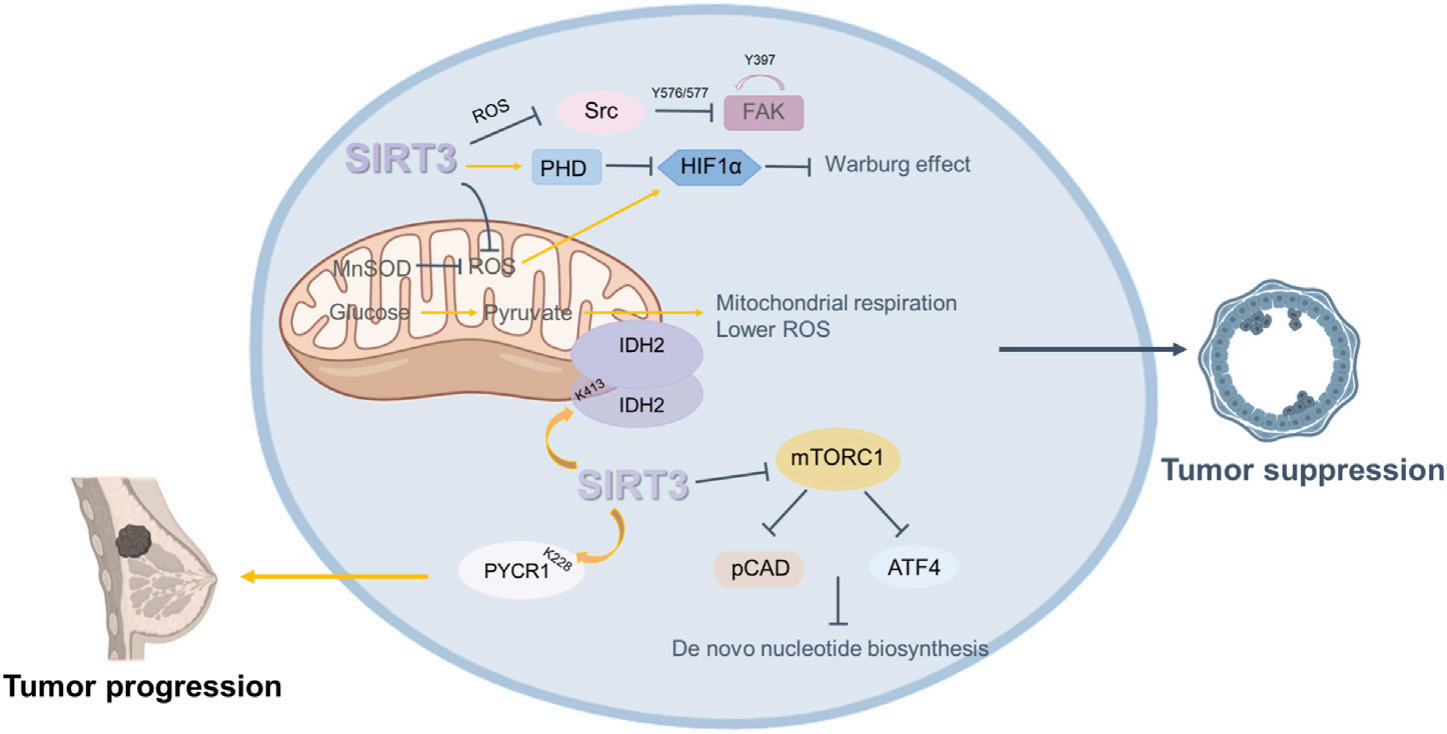
Roles of SIRT3 in breast cancer. SIRT3 plays a dual role in breast cancer. On the one hand, SIRT3 plays an oncogenic role in breast cancer. SIRT3 regulates the progression and metabolism of breast cancer cells by mediating IDH2 dimerization. SIRT3 deacetylates PYCR1 at the K228 site to modulate proline metabolism and promote the growth of MCF-7 cells. On the other hand, some studies have shown that SIRT3 plays a role in suppressing breast cancer. SIRT3 restricts breast cancer cell. Loss of SIRT3 promotes ROS production and mediates an enhanced breast tumor phenotype.

Breast cancer has ranked as the number one malignancy among women since the late 1970s, and is one of the most common types of cancer. The opinions differ among researchers concerning the roles of SIRT3 in breast cancer (Figure 4).

#### 4.1.1 Sirtuin3 as an Oncogene in Breast Cancer

Some studies have shown that SIRT3 plays a carcinogenic role in breast cancer. Within the same breast or mammary tumors, some cells are positive for SIRT3 and others are negative. However, when sorted, the SIRT3 positive cells are more aggressive and more metastatic (Kenny et al., 2017; Kenny et al., 2019). Further, the SIRT3 axis of mtUPR is important for invasion and metastasis (Kenny et al., 2019). Increased levels of SIRT3 transcription have been linked to node-positive breast cancer in divisional carcinoma biopsies (Ashraf et al., 2006). Another study showed that, among clinical characteristics, SIRT3 expression was correlated with lymph node metastasis, grade, and tumor size. Higher SIRT3 expression predicted a poorer prognosis in breast cancer patients. Clinically, tamoxifen (Tam), a selective estrogen receptor (ER) antagonist, is widely used as an adjuvant therapy in ER^+^ breast cancer treatment, but many breast cancer patients treated with Tam eventually develop drug resistance. Zhang’s team showed that SIRT3 expression was higher in Tam-resistant breast cancer cells. Knockdown of SIRT3 increased the sensitivity of Tam-resistant cells and induced apoptosis, with an increase in mitochondrial ROS and ERβ levels (Zhang et al., 2013).

There have been various studies about the oncogenic mechanism of SIRT3. The IDH2K413 acetylation mimetic weakens mitochondrial respiration and detoxification, increases ROS levels, and generates a transformed phenotype *in vitro* and *in vivo*. According to a recent study, loss of SIRT3 promotes acetylation of IDH2 at lysine 413 and subsequent dimerization of IDH 2 in breast cancer cell lines. Therefore, there is a substantial negative connection between IDH2 Lys413 acetylation and breast cancer risk and SIRT3 regulates the progression of breast cancer and the metabolism of breast cancer cells by mediating IDH2 dimerization (Zou et al., 2017). Another study showed that deacetylation of pyrroline-5-carpoxylate reductase-1(PYCR1) at the K228 site by SIRT3 can modulate proline metabolism and promote the growth of MCF-7 cells (Chen et al., 2019).

#### 4.1.2 Sirtuin3 as a Tumor Suppressor in Breast Cancer

In contrast, some studies have shown that SIRT3 plays a role in suppressing breast cancer. Kim’s team demonstrated that genetic deletion of SIRT3 causes abnormal mitochondrial physiology, destabilized genomic structure, and increased stress-induced superoxide (Kim et al., 2010). SIRT3^−/−^ MEFs with mutation of a single oncogene (Myc or Ras) altered cell metabolism and caused transformation *in vitro*, and the increased mitochondrial SOD level probably mediated the transformation of SIRT3 knockout cells. SIRT3 knockout mice spontaneously form mammary tumors, suggesting that SIRT3 acts as a tumor suppressor (Kim et al., 2010). SIRT3 protein expression is considerably lower in breast cancer tissue than in normal breast epithelium, according to the Desouki team’s research. Breast cancer patients with low SIRT3 expression have short relapse-free survival (Desouki et al., 2014).

How loss of SIRT3 promotes ROS production and mediates an enhanced tumor phenotype remains to be determined. Finley et al*.* proposed that increased ROS enhances HIF-α stabilization and promotes HIF-α target gene expression, thereby regulating the tumor phenotype. They also found that an increase in SIRT3 restricts breast cancer cell glycolysis and proliferation (Finley et al., 2011b). Furthermore, SIRT3 may also inhibit tumor growth and metastasis. It has been found that loss of SIRT3 partially enhances glutamine use in *de novo* nucleotide biosynthesis through mTORC1 activation. This finding suggests that inhibiting mTORC1 signaling and nucleotide synthesis may be a potential strategy targeting SIRT3 to inhibit tumor growth (Gonzalez Herrera et al., 2018). Haigis et al. demonstrated that upregulation of SIRT3 inhibits ROS production and represses Src/FAK signaling in metastatic breast cancer cells. The results were validated in clinical samples, which indicated that low SIRT3 levels are correlated with high metastasis rates (Lee et al., 2018).

### 4.2 Lung Cancer

Lung cancer is one of the most common cancers, and has the highest incidence and mortality rates.

#### 4.2.1 Sirtuin3 as an Oncogene in Lung Cancer

Phosphatase and tensin homolog (PTEN) is a classical tumor suppressor (Salmena et al., 2008). Defective PTEN function is a pathological mechanisms causing tumorigenesis (Song et al., 2012). Promoter methylation leads to low expression of PTEN in nearly 70% of non-small-cell lung cancer (NSCLC) cases (Marsit et al., 2005). SIRT3 is increased in PTEN-deficient NSCLC clinical samples while P53 expression is very low. SIRT3 promotes the degradation of P53 *via* the ubiquitin–proteasome pathway *via* deacetylation of P53 at lysines 320 and 382 (Xiong et al., 2018). NMNAT2, a member of the nicotinamide mononucleotide adenylyltransferase (NMNAT) enzyme family, plays critical functions in the biosynthetic pathway of NAD (NADP). The deacetylation of NMNAT2 *via* SIRT3 can enhance mitochondrial functions, mitotic entry, and cell proliferation. Thus, SIRT3 regulates cell proliferation and energy metabolism through deacetylation of NMNAT2 in lung cancer cells (Li et al., 2013).

#### 4.2.2 Sirtuin3 as a Tumor Suppressor in Lung Cancer

In contrast, Xiao et al. demonstrated that SIRT3 functions as a cancer suppressor with reduced expression in lung adenocarcinoma tissue compared with normal adjacent tissue. Increased expression of SIRT3 upregulates p21 and p53, and decreases intracellular ROS and superoxide anion levels to induce apoptosis of A549 cells (Xiao et al., 2013). The tumor suppressor gene, TP53, is mutated in the majority of human tumors, including more than 90% of small-cell lung cancer (SCLC) cases (Christensen et al., 2009). Recently, mutant-p53 (p53^Mt^) has been identified as a potential drug target for cancer therapies and a good predictor of chemoresistance in some clinical studies (Sato et al., 2018). SIRT3 can decrease p53^Mt^ stability to suppress tumors. SIRT3 induces apoptosis and necroptosis by modulating p53^Mt^ expression in SCLC (Tang et al., 2020). A recent study also reported that SIRT3 deacetylates p53^Mt^ and reduces p53 expression, inducing SCLC cell apoptosis and enhancing sensitivity to cisplatin (Guo et al., 2020a). Thus, the relationship between SIRT3 and p53^Mt^ suggests potential new approaches for SCLC treatment.

### 4.3 Hepatocellular Carcinoma

Hepatocellular carcinoma (HCC) is the most common cause of cancer-related death in developing countries.

#### 4.3.1 Sirtuin3 as a Tumor Suppressor in Hepatocellular Carcinoma

A study by Zhang’s team demonstrated that SIRT3 is a tumor suppressor in HCC (Zhang and Zhou, 2012). Upregulation of SIRT3 inhibits HCC cell proliferation and induces apoptosis *via* regulation of the ERK, p38, JNK pathways and altered NAD^+^ levels. Furthermore, increased expression of SIRT3 upregulates p53 levels by attenuating Mdm2-mediated p53 degradation, suggesting that SIRT3 may play a critical role in inhibiting HCC progression (Zhang and Zhou, 2012).

### 4.4 Gastric Cancer

#### 4.4.1 Sirtuin3 as an Oncogene in Gastric Cancer

SIRT3, involved in mitochondrial metabolic homeostasis in gastric cancer, is considered a cancer-promoting factor. It has been reported that SIRT3 can promote lactate dehydrogenase A (LDHA) deacetylation and activation to enhance glycolysis and proliferation in gastric cancer (Cui et al., 2015).

#### 4.4.2 Sirtuin3 as a Tumor Suppressor in Gastric Cancer

However, another study showed that SIRT3 can function as a tumor suppressor in gastric cancer. The multivariate analysis reported that SIRT3 could be an independent biomarker for the prediction of gastric cancer prognosis (Huang et al., 2014). The expression of SIRT3 is negatively related to clinicopathological variables, including tumor invasion, differentiation, and stage. Moreover, SIRT3 knockdown in MGC-803 cells upregulates the expression of HIF-1α (Yang et al., 2014a). It was reported that downregulation of Notch-1 by SIRT3 inhibits the proliferation of gastric cancer cells (Wang et al., 2015).

### 4.5 Colon Cancer

#### 4.5.1 Sirtuin3 as an Oncogene in Colon Cancer

SIRT3 is highly expressed in colorectal cancer, and this high expression is correlated with tumor stage and lymph node metastasis in colon cancer. Higher SIRT3 expression might shorten the colon cancer-specific survival and the overall survival of patients (Liu et al., 2014). Thus, SIRT3 may be a marker for colon cancer. Serine hydroxymethyl transferase 2 (SHMT2) is reported to be acetylated at the K95 site in colorectal cancer cells. Functional studies indicated that SHMT2-K95-Ac inhibits SHMT2 enzymatic activity by disrupting the functional tetramer structure and promoting its degradation through macroautophagy. SIRT3 is responsible for the deacetylation of SHMT2 and thereby promotes colorectal tumorigenesis (Wei et al., 2018).

Some previous studies have found SIRT3 can maintain process involved in mitochondrial homeostasis, including mitochondrial biogenesis and function. Knockdown of SIRT3 results in downregulation of genes that play roles in mitochondrial biogenesis and function, including NRF1, TEAM, and MTSSB. Knockdown of SIRT3 also downregulates OXPHOS protein levels, COX activity, mitochondrial ATP production, and maximal respiration capacity but increases LDH activity (Torrens-Mas et al., 2019). Loss of SIRT3 triggers activation of mitochondrial fission through the Akt/PTEN pathway and modulates inhibition of colorectal cancer cell survival, growth, and mobility (Wang et al., 2018). In addition, SIRT3 has been demonstrated to be an independent prognostic factor in colon cancer. SIRT3 facilitates chemoresistance in colon cancer cells by regulating SOD2 and PGC-1α (Paku et al., 2021). Therefore, SIRT3 may be a therapeutic and novel target for inhibiting colon cancer.

#### 4.5.2 Sirtuin3 as a Tumor Suppressor in Colon Cancer

Notably, there is a novel antitumor mechanism. Mitochondrial pyruvate carrier 1 (MPC1) has been reported to be downregulated in colon cancer and is associated with a poor prognosis (Schell et al., 2014). Liang et al. found that SIRT3 binds to MPC1 and deacetylates it to inhibit colon cancer cell growth driven by high glucose (Liang et al., 2015).

### 4.6 Prostate Cancer

Prostate cancer is the second leading cause of cancer-related deaths in men.

#### 4.6.1 Sirtuin3 as an Oncogene in Prostate Cancer

Receptor-interacting serine/threonine-protein kinase 3 (RIPK3) is a key regulator activating necroptosis and the innate immune response. The phosphorylation of RIPK3 can be suppressed by increased expression of SIRT3 and SIRT6 in prostate cancer, which induces the dysregulation of necroptosis. This study suggested that SIRT3 and SIRT6 promote tumor progression (Fu et al., 2020).

#### 4.6.2 Sirtuin3 as a Tumor Suppressor in Prostate Cancer

Inconsistent with the above reports, upregulation of SIRT3 inhibits prostate cancer cell growth by repressing the PI3K-AKT pathway and c-myc (Quan et al., 2015a). Moreover, SIRT3 inhibits epithelial-mesenchymal transition (EMT) and migration by regulating FOXO3A expression and suppressing the Wnt/β-catenin pathway in prostate cancer (Li et al., 2018).

### 4.7 Ovarian Cancer

Ovarian cancer is the leading cause of gynecological cancer-associated deaths.

#### 4.7.1 Sirtuin3 as a Tumor Suppressor in Ovarian Cancer

Clinically, preventing metastasis is a major obstacle to controlling ovarian cancer. It was reported that SIRT3 is critical for the inhibition of ovarian cancer invasion and metastasis. Mechanistically, SIRT3 suppresses Twist to inhibit epithelial-mesenchymal transition in ovarian cancer (Dong et al., 2016). SIRT3 is highly expressed in anchorage-independent ovarian cancer cells. Additionally, SIRT3 regulates mitochondrial ROS levels by activating SOD2, and glycolysis is upregulated by SIRT3 knockdown in anchorage-independent ovarian cancer cells (Kim et al., 2020).

### 4.8 Esophageal Squamous Cell Carcinoma

#### 4.8.1 Sirtuin3 as an Oncogene in Esophageal Squamous Cell Carcinoma

SIRT3 is highly expressed in esophageal carcinoma and is related to short survival time in esophageal cancer patients (Zhao et al., 2013). Downregulation of SIRT3 significantly inhibits the proliferation and induces the apoptosis of EC9706 cells. Loss of SIRT3 upregulates p21 and Bax expression but reduces Bcl-2 expression (Yang et al., 2014b).

### 4.9 Bladder Cancer

Bladder cancer is a common malignant tumor in the urinary system.

#### 4.9.1 Sirtuin3 as an Oncogene in Bladder Cancer

The function of p53 is to guide cell cycle arrest, cell aging and apoptosis. In EJ bladder carcinoma cells that express wild-type p53, SIRT3 inhibits p53-mediated growth arrest. Mechanistically, BAG-2 (BCL2-associated athanogene protein) interacts with p53, to stabilize acetylated p53 and deacetylate it; SIRT3 promotes tumor growth (Li et al., 2010).

### 4.10 Diffuse Large B-Cell Lymphomas

Diffuse large B-cell lymphomas (DLBCLs) are highly genetically heterogeneous and aggressive neoplasms.

#### 4.10.1 Sirtuin3 as an Oncogene in Diffuse Large B-Cell Lymphomas

A recent study showed that upregulation of SIRT3 was associated with an unfavorable outcome in DLBCL. Knockout of SIRT3 inhibited lymphomagenesis and prolonged survival in VavP-Bcl2 mice without impairing the formation of normal germinal centers. Moreover, SIRT3 is necessary to maintain the TCA cycle in DLBCLs. Depletion of SIRT3 reduces acetyl-CoA pools and induces GDH hyperacetylation to impair glutamine flux to the TCA cycle. Because of the impairment of the TCA cycle, cells need to gain metabolic precursors in other ways. Thus, depletion of SIRT3 leads to induction of autophagy and cell death (Canepa et al., 2019).

Indeed, autophagy can recover degraded intracellular nutrients to maintain cell metabolism and alleviate cell damage (Kimmelman and White, 2017). However, the results of this study showed that autophagy, caused by SIRT3 downregulation promotes it as a tumor suppressor (Canepa et al., 2019). Autophagy may play different roles in cancer depending upon the type of degraded proteins affected by autophagy. Autophagy may aid in tumor suppression degrading of the proteins important to metabolic pathways and DNA replication (Mathew et al., 2014).

### 4.11 Head and Neck Cancer

Oral squamous cell carcinoma (OSCC) accounts for approximately 90% of all oral malignancies.

#### 4.11.1 Sirtuin3 as an Oncogene in Oral Squamous Cell Carcinoma

SIRT3 acts as an oncogene in OSCC cells. SIRT3 is highly expressed in OSCC cells, and downregulation of SIRT3 inhibits cell proliferation and enhances sensitivity to radiation and cisplatin treatment (Alhazzazi et al., 2011).

#### 4.11.2 Sirtuin3 as a Tumor Suppressor in Oral Squamous Cell Carcinoma

Paradoxically, Chen et al. demonstrated that the expression of SIRT3 is higher in OSCC than in normal human oral keratinocytes, but its enzymatic deacetylation activity is downregulated. They researcher found a SIRT3 mutation near the active deacetylase site, that reduces the overall deacetylase activity. Upregulation of SIRT3 inhibited OSCC cell growth and decreased basal ROS levels in OSCC cell lines (Chen et al., 2013). Therefore, SIRT3 can act as a tumor suppressor in OSCC cell lines.

#### 4.11.3 Sirtuin3 as an Oncogene in Tongue Cancer

Zhou et al. found that knockout of SIRT3 promotes tongue cancer cell apoptosis. SIRT3 knockdown activated the c-Jun N-terminal kinase (JNK) signaling pathway to modulate Fis1 expression. Subsequently, upregulation of Fis1 promoted mitochondrial fission and stress, leading to cell apoptosis. In summary, this study revealed that SIRT3 acts as an oncogene in tongue cancer *via* regulation of the JNK-Fis1 axis.

### 4.12 Cervical Cancer

Cervical cancer is one of the most common gynecologic malignant tumors.

#### 4.12.1 Sirtuin3 as an Oncogene in Cervical Cancer

SIRT3-mediated regulation of fatty acid synthesis is critical for the proliferation and metastasis of cervical cancer cells. SIRT3 can deacetylate and activate acetyl CoA carboxylase 1 (ACC1) to promote fatty acid synthesis, suggesting an oncogenic role of SIRT3 (Xu et al., 2020).

### 4.13 Melanoma

Melanoma is the most aggressive skin cancer. Existing prevention and therapies fail to effectively manage the incidence and fatality of melanoma, making it a critical clinical problem.

#### 4.13.1 Sirtuin3 as an Oncogene in Melanoma

SIRT3 is highly expressed in multiple human melanoma cells. SIRT3 knockdown inhibits cell proliferation and survival, inducing senescence by upregulating SA-β-Gal activity (George et al., 2016). In addition, it was reported that mutations in the TP53 gene can occur in 35% of sporadic skin cancers (Olivier et al., 2010). Most are missense mutations leading to dysfunction of the p53 protein (Yamamoto and Iwakuma, 2018). Torrens-Mas et al. indicated that Tp53 mutation activates the SIRT3-MnSOD axis to regulate ROS production in melanoma (Torrens-Mas et al., 2020).

### 4.14 Dual Role of SIRT3 in Cancer

**FIGURE 5:**
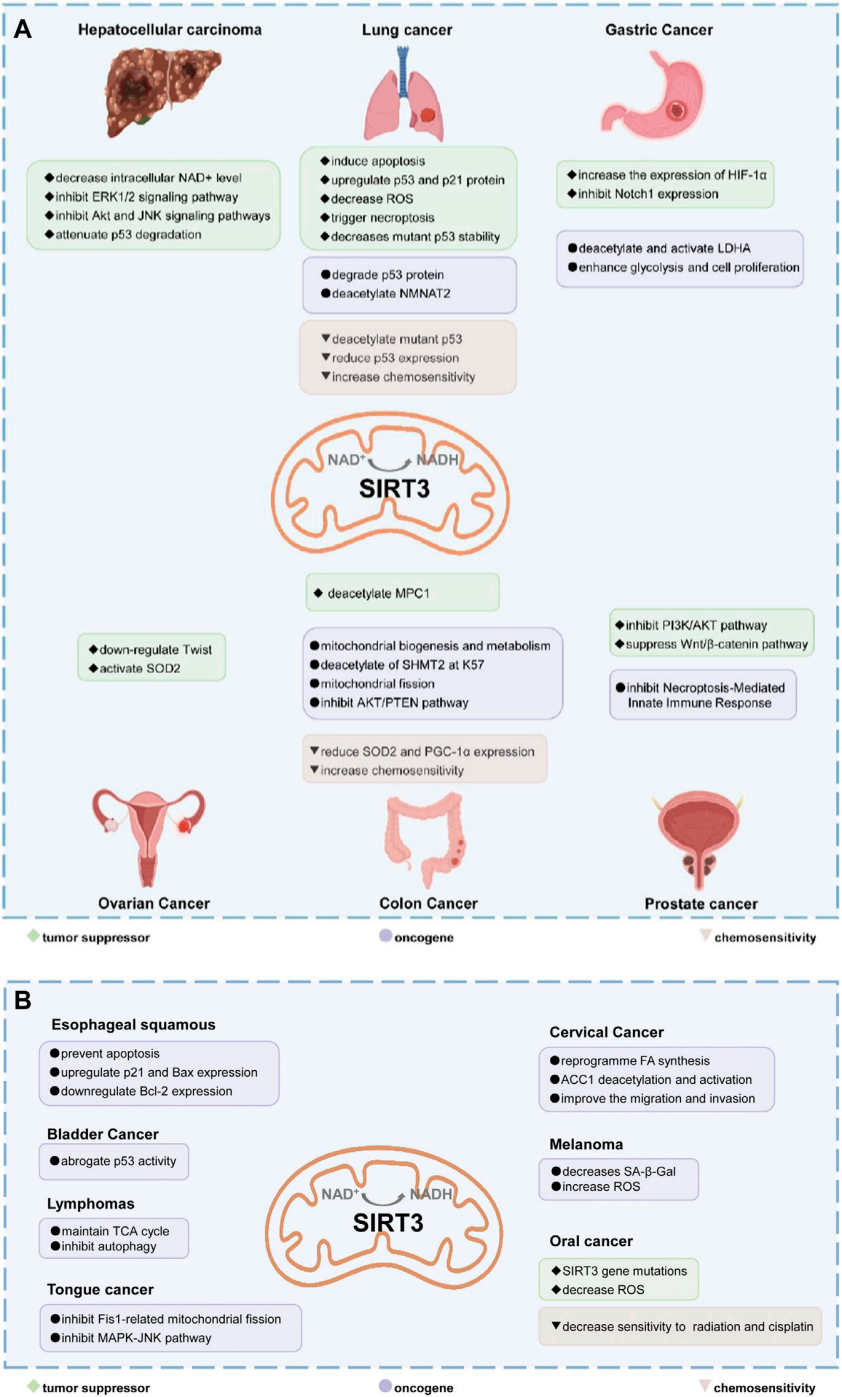
The dual role of SIRT3 in different cancers. **(A,B)** In most cancers, SIRT3 plays a tumor suppressor role. SIRT3 can maintain the stability of the genome and inhibit carcinogenesis. SIRT3 inhibits the Warburg effect in cancer to inhibit tumor development. In addition, SIRT3 can inhibit tumor cell proliferation and metastasis, and induce apoptosis and autophagy. However, in some cancers, such as lung cancer, gastric cancer, colon cancer, prostate cancer, and oral cancer, SIRT3 acts as an oncogene by promoting proliferation and metastasis through deacetylation of specific substrates. Text in green with diamond bullets indicates SIRT3 is a tumor suppressor in those cancers. The purple text with the circular bullets indicates that SIRT3 is an oncogene in those cancers. The brown text with triangular bullets indicates that SIRT3 can increase sensitivity to chemoresistance in those cancers.

In summary, the function of SIRT3 varies in different cancers, with SIRT3 having cell-and tumor-type specificity, and its role may largely depend on the cellular conditions (Figure 5). On the one hand, SIRT3 inhibits the Warburg effect, tumor cell proliferation and metastasis to serve as a tumor suppressor. While on the other hand, SIRT3 promotes cell metabolism and deacetylates specific substrates to play a carcinogenic role. Tumors undergo metabolic reprogramming and activate different metabolic modes for survival. Most tumors are dependent on aerobic glycolysis, while others are dependent on oxidative phosphorylation. SIRT3 can promote oxidative phosphorylation and inhibit glycolysis in the regulation of mitochondrial metabolism, which may be one of the reasons why it plays a dual role in cancer. In addition, deacetylated substrates of SIRT3 may play different regulatory roles, thus affecting the occurrence and development of tumors.

SIRT3 can maintain mitochondrial homeostasis, which is necessary for survival Therefore, SIRT3 is regulated in numerous ways. Cancer cells usually possess higher ROS levels than normal cells, and the upregulation of SIRT3 in tumors may be to reduce oxidative damage and enhance the mitochondrial stress defense system in some cases. Higher ROS levels may promote cancer progression and chemotherapy resistance. However, some anticancer approaches rely on an ability to promote ROS production and lead to cell death to overcome drug resistance. SIRT3 is a potential target for new therapies for treating cancer.

Overall, the dual role of SIRT3 may render the utility of SIRT3 as a target of cancer treatment uncertain. It is important to note that SIRT3-specific treatment is very important in the research of different types of cancer. Understanding the critical role of SIRT3 in different cancer types may help to enhance our knowledge of carcinogenic and anticancer effects. Further characterization of the mitochondrial substrates of SIRT3 will provide important strategies for future applications (Chen et al., 2014).

## 5 Targeting Sirtuin3 in Cancer Therapeutics

The function of SIRT3 in aging, neurological disease, cancer, and stress resistance has drawn much attention. By reversible protein lysine deacetylation, SIRT3 can regulate mitochondrial activity and biosynthetic processes such as the TCA cycle, oxidative stress, glucose, fatty acid metabolism, and apoptosis. The therapeutic potential of SIRT3 in multiple cancers makes it an attractive drug target. The critical biological functions of SIRT3 have sparked research into small molecule modulators that can control its activity (Villalba and Alcaín, 2012). Currently, there is no specific and selective SIRT3 activator or inhibitor due to the conserved molecular structure of the SIRTs family and the mode of SIRT3 activation. In this section, recent advances in the discovery of SIRT3 activators and inhibitors are reviewed.

### 5.1 Activators of Sirtuin3

SIRT3 regulates a range of physiological functions and plays a preventive role in some disorders by maintaining mitochondrial health. Therefore, it is necessary and meaningful to develop SIRT3 activators. Herein, we systematically summarize potential SIRT3 activators (Table 2). Resveratrol and honokiol are the most classic activators of SIRT3. Resveratrol is a natural polyphenolic antioxidant originally identified as a phytoalexin. Desquiret-Dumas et al. demonstrated that resveratrol promotes SIRT3 activation and gives rise to a complex I-dependent increase in NADH oxidation (Desquiret-Dumas et al., 2013). By boosting SIRT3 enrichment and subsequently elevating the FOXO3a-mediated expression of the mitochondria-encoded genes CO1, Cytb, ATP6, ND2, and ND5, resveratrol significantly lowers mtROS production, resulting in higher complex I activity and ATP synthesis (Zhou et al., 2014). As a result, resveratrol is considered a SIRT3 activator. Honokiol is a natural biphenolic chemical found in magnolia tree bark. Honokiol inhibits the hypertrophic response and pressure overload cardiac hypertrophy by activating SIRT3 (Pillai et al., 2015).

**TABLE 2:** Activator of SIRT3.

Compd name in the publication	Chemical structure	Kd value (SIRT3)	Effect on other SIRTs	References
Resveratrol	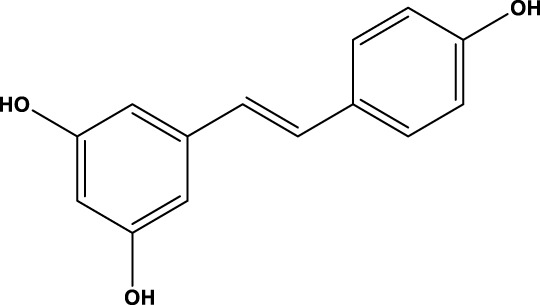	445 μM	SIRT1	Desquiret-Dumas et al. (2013); Zhou et al. (2014)
Honokiol	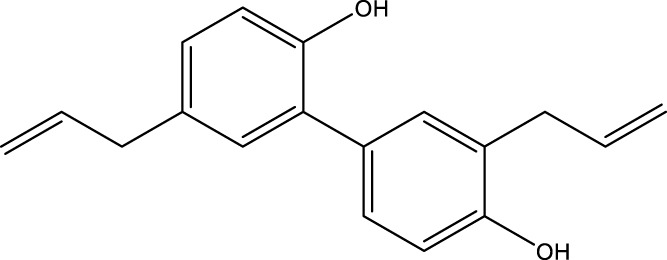	-	-	Pillai et al. (2015)
Oroxylin A	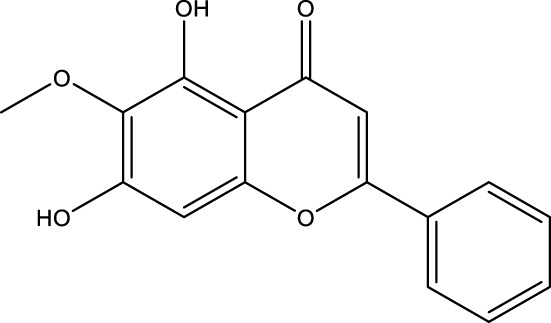	-	-	Wei et al. (2013)
Adjudin	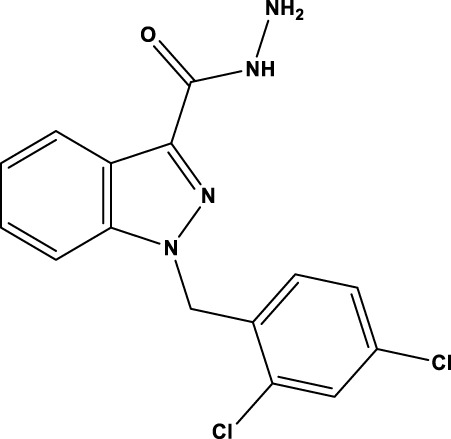	-	-	Quan et al. (2015b)
Pyrroloquinoline quinone	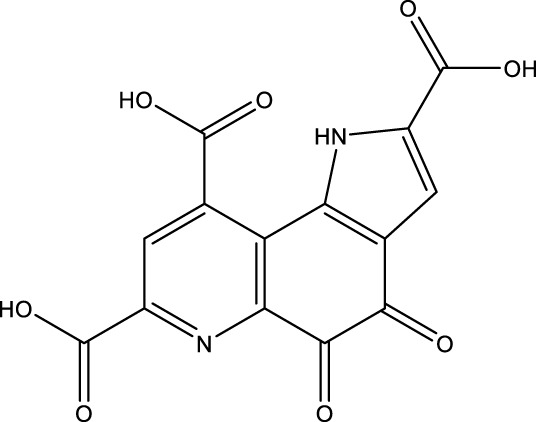	-	SIRT1	Zhang et al. (2015)
Dihydromyricetin	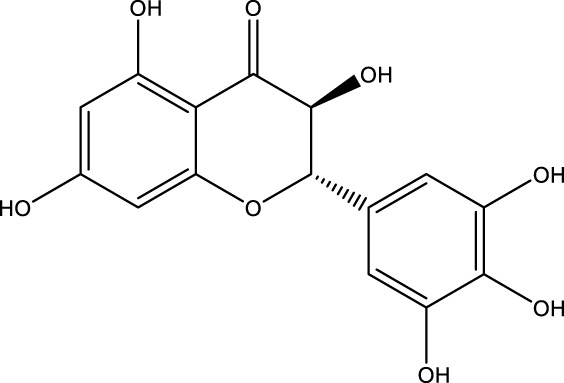	-	SIRT1	Liu et al. (2016); Woo et al. (2012)
Silybin	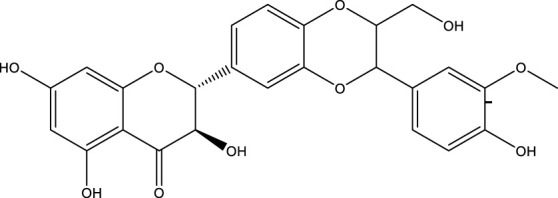	-	SIRT2	Li et al. (2017)
Melatonin	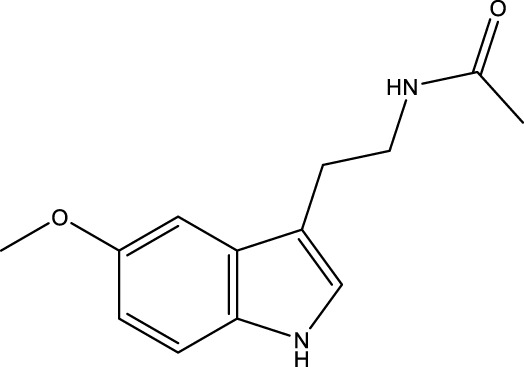	-	SIRT1	Zhai et al. (2017)
Polydatin	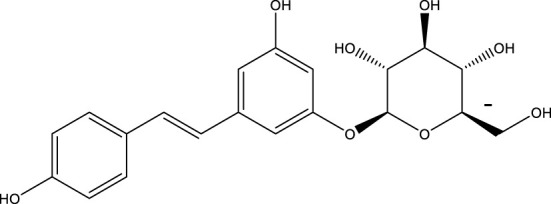	-	SIRT1	Zhang et al. (2017)
C12	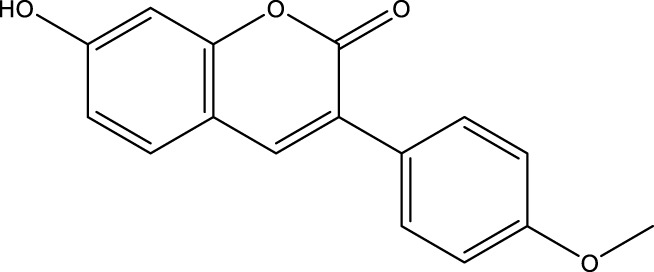	3.9 μM	-	Lu et al. (2017)
Berberine	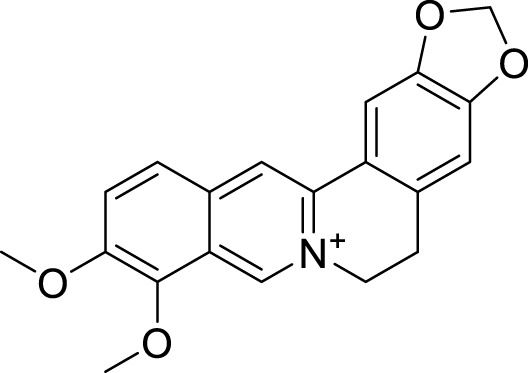	-	SIRT1	Coelho et al. (2017)
Sesamin	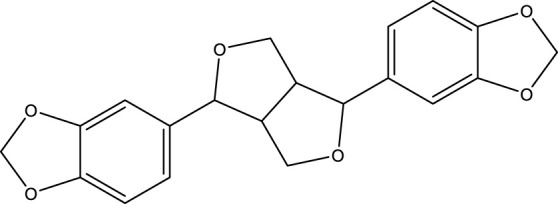	-	SIRT1	Fan et al. (2017)
PNU-282987	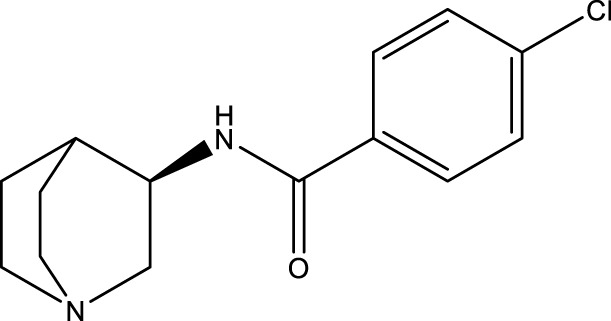	-	-	Li et al. (2019a)
licoisoflavone A	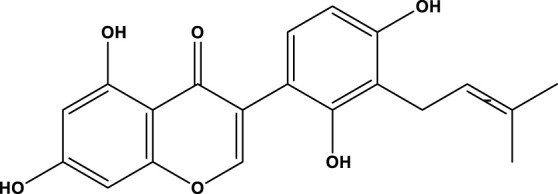	-	-	Guo et al. (2020b)
pomegraniin A	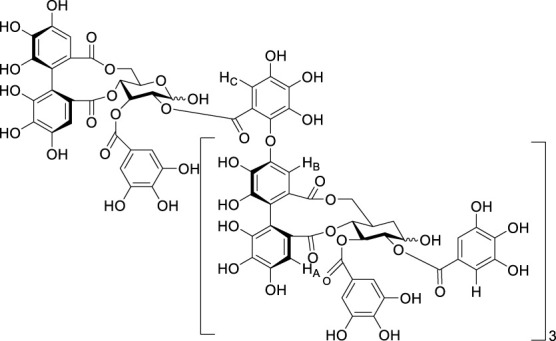	-		Ito et al. (2014); Zhao et al. (2016)
Liraglutide	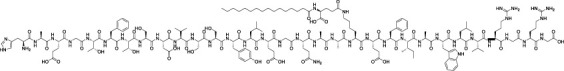	-		Li et al. (2019b)

Oroxylin A (OA) is a flavonoid isolated from Scutellaria root that has antiviral, anti-inflammatory, antioxidant, and anticancer properties. By upregulating the SIRT3 level, oroxylin A promotes the dissociation of HK II from the mitochondria and hinders glycolysis in breast cancer (Wei et al., 2013). Adjudin, an analog of lonidamine, has been identified as a potential male contraceptive. Quan et al. found that adjudin may be a novel otoprotective agent that protects hair cells from ototoxic stimuli by increasing SIRT3 levels (Quan et al., 2015b). Pyrroloquinoline quinone (PQQ), an aromatic heterocyclic anionic orthoquinone, can act as an activator of SIRT1 and SIRT3 and is a promising therapeutic agent for metabolic illnesses (Zhang et al., 2015). The flavonoid dihydromyricetin, a natural antioxidant with beneficial qualities, can be extracted from the Ampelopsis grossedentata plant (Liu et al., 2016). By increasing mitochondrial biogenesis *via* SIRT3-mediated FOXO3 deacetylation, dihydromyricetin may help to protect neural function (Woo et al., 2012).

Silybin is a polyphenolic flavonoid with antioxidative and antitumor properties. Silybin is a SIRT3 activator that protects tubular cells against cisplatin-induced apoptosis and from acute renal injury by enhancing mitochondrial function (Li et al., 2017). Melatonin, primarily produced by the pineal gland, is well known for its antioxidant and free radical scavenging properties. Melatonin therapy reduces oxidative stress and apoptosis in myocardial ischemia–reperfusion (MI/R) damage by activating the SIRT3 signaling pathway (Zhai et al., 2017). Polydatin is a monocrystalline and polyphenolic drug derived from Polygonum cuspidatum. Zhang et al. discovered that polydatin improves cardiac function after MI and targets SIRT3 to regulate autophagy, apoptosis, and mitochondrial biogenesis (Zhang et al., 2017).

Lu et al. created a mutant MnSOD with N-acetyllysine (AcK) at Lys68 to examine the effect of Lys68 acetylation on MnSOD activity. They discovered a novel SIRT3 activator, C12 (7-hydroxy-3-(4′-methoxyphenyl) coumarin), which has a strong affinity for SIRT3 and can boost MnSOD deacetylation and activation, based on an assay they devised for SIRT3-mediated deacetylation of MnSODK68AcK. Isothermal titration calorimetry (ITC) assays showed that C12 binds to SIRT3 with a Kd value of 3.9 µM, which is lower than resveratrol’s Kd value of 445 µM (Lu et al., 2017). Berberine, a natural compound from traditional Chinese medicine, attenuates DOX-induced cardiotoxicity through SIRT3 (Coelho et al., 2017). Sesamin, a well-known antioxidant derived from sesame seeds, has been shown to improve cardiac function and prevent ventricular hypertrophy by activating the SIRT3/ROS pathway. Fan et al. confirmed that SIRT3 is a target of sesamin (Fan et al., 2017). PNU-282987, a selective alpha 7 nicotinic acetylcholine receptor (α7nAChR) agonist, enhances mitochondrial SIRT3 deacetylase activity but does not regulate SIRT3 protein expression. Moreover, PNU-282987 enhances the deacetylation of mitochondrial FOXO3 (Li et al., 2019a). Licoisoflavone A, a main active component from Tongmaiyangxin, was shown to prevent the hypertrophic response of cardiomyocytes by upregulating SIRT3. Therefore, Guo et al. suggested that licoisoflavone A can be a potential SIRT3 activator with therapeutic potential for cardiac hypertrophy (Guo et al., 2020b). Pomegraniin A (Ito et al., 2014) can improve intestinal injury caused by hemorrhagic shock *via* reducing ROS *via* SIRT3-dependent SOD2 activation in Caco-2 cells (Zhao et al., 2016). Thus, pomegraniin A is a potential activator of SIRT3. Liraglutide is a type of glucagon-like peptide-1 agonist. It can protect renal mesangial cells against mitochondrial apoptosis caused by hyperglycemia by upregulating SIRT3 expression and activating the ERK-YAP signaling pathway (Li et al., 2019b).

Most of these potential activators of SIRT3 are natural products and antioxidants suggesting that SIRT3 is a key regulator of oxidative stress and a ROS scavenger. Compounds with good antioxidant effects may be favorable for SIRT3 activator screening. However, for most of these compounds, more evidence is necessary to explore how they can activate SIRT3 and verify whether the interaction is direct. Among the potential activators of SIRT3 summarized above, C12 is a small-molecule compound with better affinity. Therefore, further study of the detailed mechanism of C12 may aid the development of a potential activator with better selectivity and efficacy.

### 5.2 Inhibitors of Sirtuin3

All SIRTs share a largely conserved catalytic core, which leads to the limited potency and isoform selectivity of most SIRTs inhibitors identified to date. The action pattern of SIRT3 has not been fully elucidated, which limits the development of SIRT3 inhibitors. Here, we will review the SIRT3 inhibitors with relatively better selectivity and specificity (Table 3). In the Galli team’s study, they identified 3-triazolylpyridine(3-TYP) as the first inhibitor of SIRT3, which was called compound 2 in the article. 3-TYP is a selective and effective SIRT3 inhibitor with an IC50 of 16 nM, making it stronger than SIRT1 (IC50: 88 nM) and SIRT2 (IC50: 92 nM) inhibitors. 3-TYP can decrease ATP levels and increase superoxide generation, and the biological effects of 3-TYP are consistent with those effects seen in SIRT3 knockout mice. However, 3-TYP is a simple bioisosteric analog of nicotinamide, therefore, it may interact with other proteins and show off-target activity. Regardless, 3-TYP provides a basis for agents with a greater degree of chemical modification and the identification of more potent and selective SIRTs inhibitors (Galli et al., 2012). 3-TYP inhibits melatonin-enhanced SIRT3 activity without altering SIRT3 expression (Pi et al., 2015), and reduces SIRT3 activity while increasing SOD2 acetylation. Furthermore, 3-TYP reduces left ventricular ejection fraction (LVEF) and left ventricular fractional shortening (LVFS) following reperfusion, reducing the cardioprotective effects of melatonin (Zhai et al., 2017). SRT1720 was first found to be a human SIRT1 activator, however, it also inhibits human SIRT3 activity. The crystal structure of SIRT3 with the SRT1720 complex and an NAD^+^ analog suggests that SRT1720 partially occupies the acetyl-Lys binding site (Nguyen et al., 2013a).

**TABLE 3:** Inhibitor of SIRT3.

Compd name in publication	Chemical structure	Cellular IC_50_ (SIRT3)	References
3-TYP	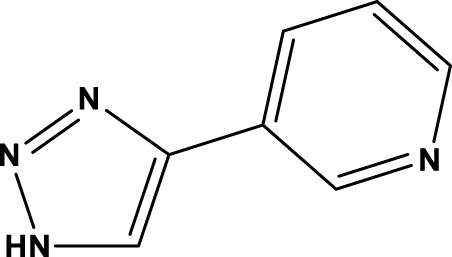	16 nM	Galli et al. (2012); Pi et al. (2015); Zhai et al. (2017)
SRT1720	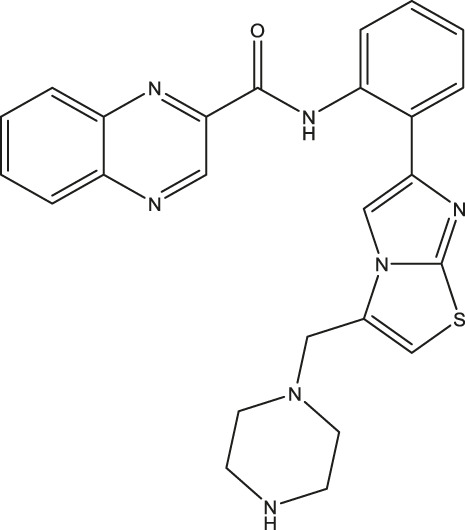	-	Nguyen et al. (2013a)
11c (Disch et al.)	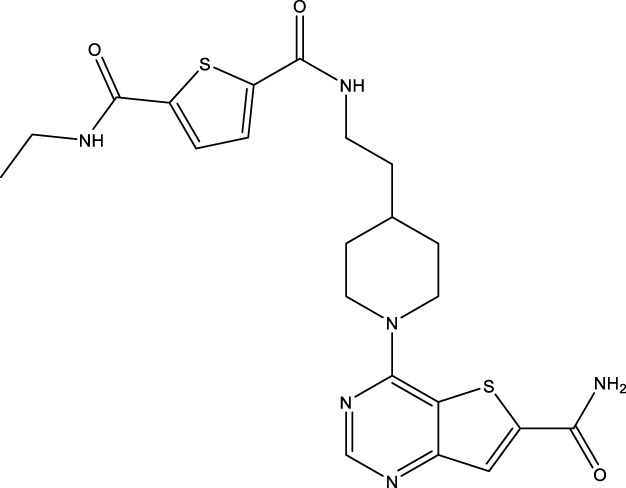	4.0 nM	Disch et al. (2013)
28 (Disch et al.)	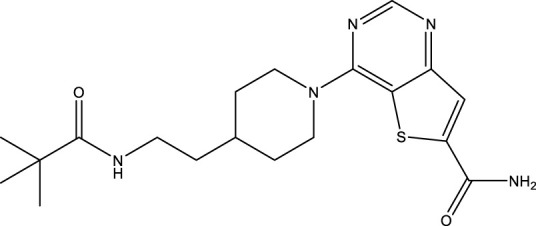	33 nM	Disch et al. (2013)
31 (Disch et al.)	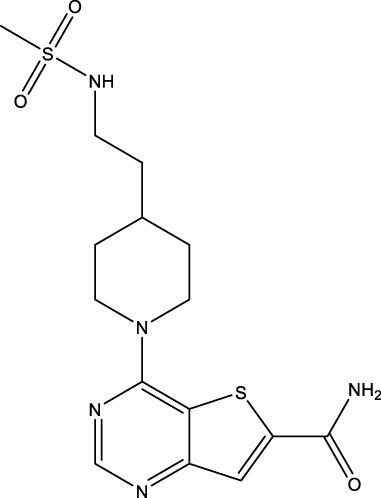	7 nM	Disch et al. (2013)
4-Bromo-resveratrol	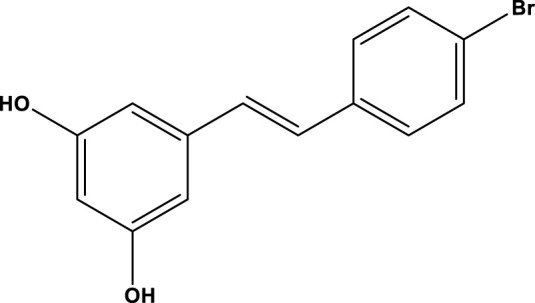	143.0 ± 3.6 μm	Nguyen et al. (2013b)
compound 8	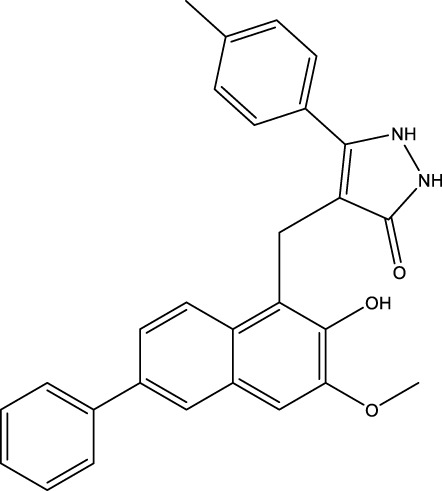	6 μm	Mahajan et al. (2014)
7 (Kokkonen et al.)	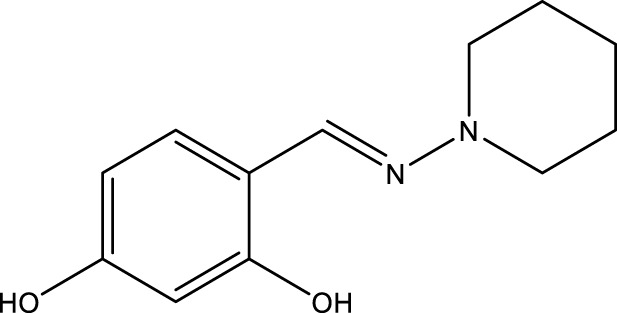	32.0 ± 4.0 μm	Kokkonen et al. (2015)
12 (Kokkonen et al.)	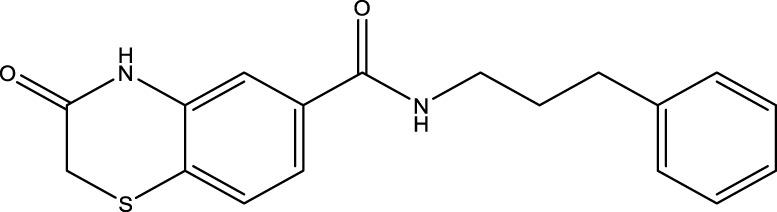	40.0 ± 5.7 μm	Kokkonen et al. (2015)
13 (Kokkonen et al.)	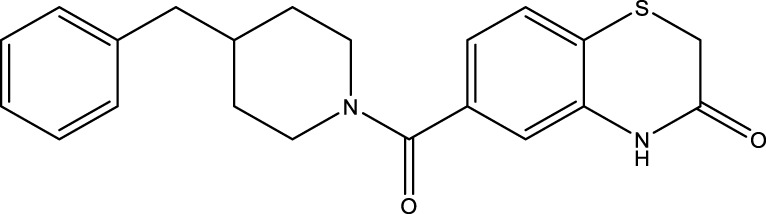	38.0 ± 1.4 μm	Kokkonen et al. (2015)
**7** (Chen et al.)	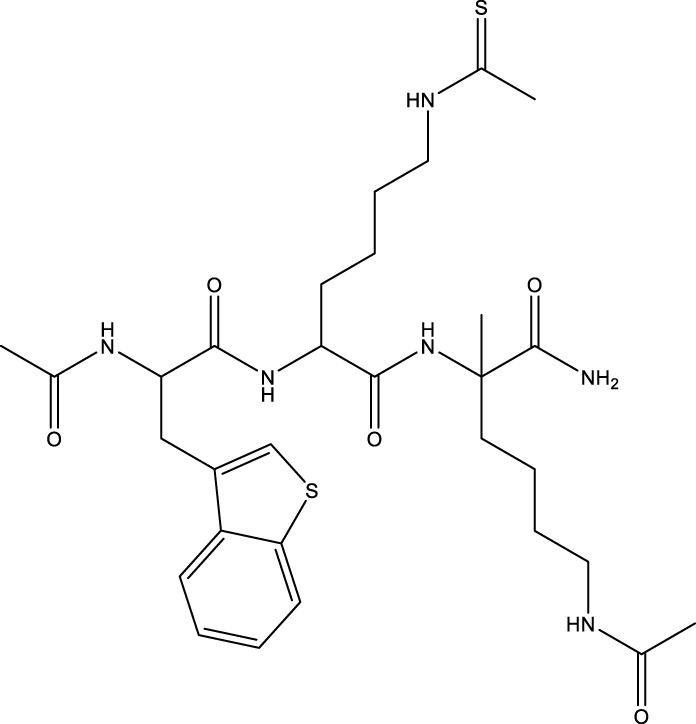	1.44 μm	Chen et al. (2015)
9 (Chen et al.)	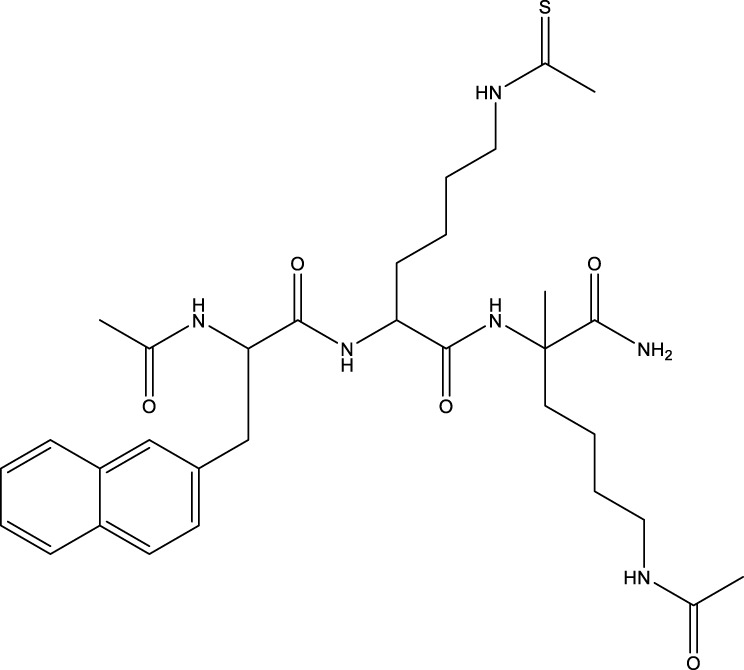	1.29 μm	Chen et al. (2015)
19 (Chen et al.)	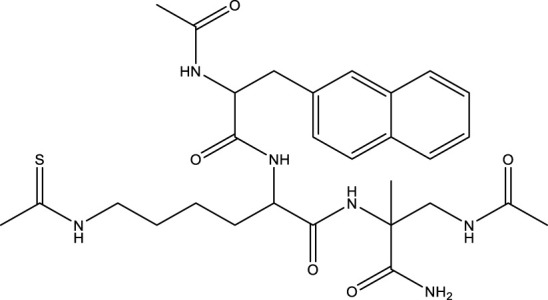	1.91 ± 0.12 μm	Chen et al. (2015)
LC-0296	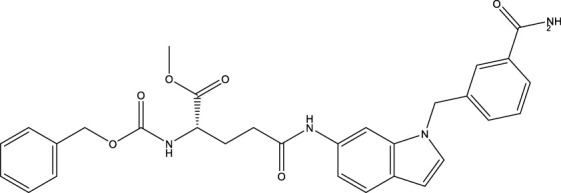	3.6 μm	Alhazzazi et al. (2016)
2-methoxyestradiol	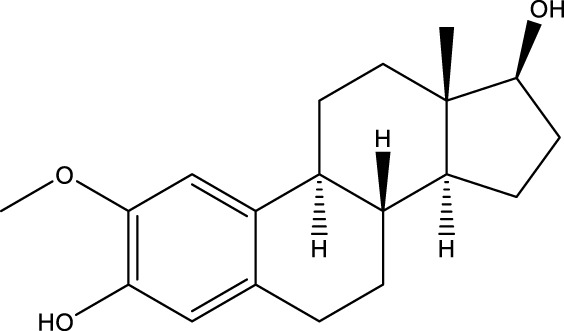	-	Gorska-Ponikowska et al. (2018)
YC8-02	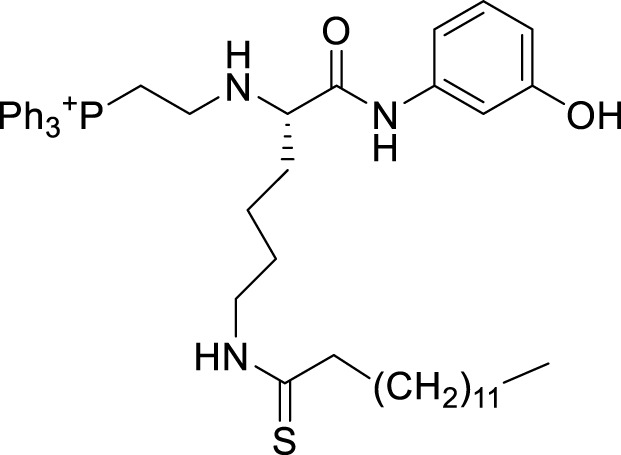	0.53 μm	Canepa et al. (2019)

Compound 11c and the truncated analogs 28 and 31 were recognized by encoded library technology, and represent important advances in current SIRTs inhibitors (Disch et al., 2013). The molecular mechanics/generalized Born surface area (MM-GBSA) method has been used as a predictive model for developing a series of published SIRT1-3 inhibitors (Disch et al., 2013). 4′-Bromo-resveratrol was validated as a novel potent SIRT3 inhibitor and effectively inhibits SIRT3 activity at 0.2 mM. The crystal structures of human SIRT3 and peptide complexes demonstrate that there are two compound binding sites for 4′-bromo-resveratrol on SIRT3. One is an internal site that overlaps with the active site and causes effective inhibition. The other one is located on the surface of the SIRT3 protein and is connected to the active peptide binding site ring by two helices (Nguyen et al., 2013b).

Compound 8, prepared by the Mehajan team, is a potent SIRT3 inhibitor (IC_50_ at 6 μM), that has relatively low binding with SIRT1 (IC_50_ at 41 μM) and SIRT2(IC_50_ at 32 μM) (Mahajan et al., 2014). Halgren et al. identified a potential binding slot in the zinc-binding domain comprised of the residues Phe186, Gln260, Asp290, and Glu296 using a SiteMap v. 2.6 feature (Halgren, 2009). A few years later, scientists used this putative binding region of SIRTs to discover new SIRTs inhibitors by screening the ZINC database, the study discovered three moderately potent SIRTs inhibitors (7, 12, 13). Further research found that the inhibitors had two new scaffolds, and the smaller scaffold is regarded as a promising fragment (Kokkonen et al., 2015). However, it is worth noting that the development of SIRT3 inhibitors is still in its infancy, and the most important SIRT3 inhibitors reported in the literature are compounds identified by Disch et al. (2013). However, the SIRT3 inhibitory selectivity of these compounds is moderate. In Chen’s study, the researcher found that Compounds 7, 9, 19 showed significantly enhanced SIRT3 inhibitory selectivity: they showed a 10-fold greater inhibition of SIRT3 than of SIRT1 or SIRT2. In addition, Compound 19 showed higher selectivity for SIRT3 than SIRT5/6. Thus, the identification of these compounds is an excellent starting point for the development more effective and selective SIRT3 inhibitors based on catalytic activity (Chen et al., 2015). Butyrate is the final product of intestinal microbial fermentation of dietary fiber, and its anti-tumor effects are still unclear. In recent years, butyrate has been recognized as a SIRT3 inhibitor promoting cancer cell apoptosis and treatment with butyrate inhibited the ability of SIRT3 to deacetylate a synthetic acetylated pyruvate dehydrogenase E1α subunit (PDHA1) peptide containing K336 (Xu et al., 2017). In another study, researchers developed a novel SIRT3 inhibitor LC-0296, with selective inhibition of SIRT3 enzyme activity (IC_50_ at 3.6 μM), with the IC_50_ values for SIRT1 and SIRT2 being approximately ∼20- and 10-fold lower than that for SIRT3 (Alhazzazi et al., 2016). 2-Methoxyestradiol (2-ME), a potent anticancer agent, has also been shown to be an effective inhibitor of SIRT3 that functions by binding to the typical inhibitor binding site and allosteric site (Gorska-Ponikowska et al., 2018). A new study proposed that SIRT3 could be an attractive drug target, because of its important role in DLBCL cell proliferation and survival. In that study, Li et al. developed a compound called YC8-02 (IC_50_ at 0.53 μM) that could inhibit SIRT3 at nanomolar concentrations in biochemical enzymatic assays (Canepa et al., 2019).

However, there are no reports of clinical trials of SIRT3 inhibitors. In addition to developing compounds targeting the deacetylation active site of SIRT3, some researchers have focused on targeting the demyristoylation and depalmitoylation sites. For example, an improved fluorogenic assay was established by Chiang et al. using a new myristoyl peptide with C-terminal 7-amino-4-methylcoumarin (AMC) as a substrate, which accelerates the high-throughput screening of SIRT3 modulators (Chiang and Lin, 2016). Gai et al. suggested that SIRT1-3 exhibit enzymatic activities on myristoylated and palmitoylated peptides, but their catalytic efficiencies are different. These theoretical findings indicate that allosteric sites can be used as targets for designing isoform-selective inhibitors (Gai et al., 2016). In summary, new strategies have promise for developing SIRT3 modulators with excellent affinity, specificity, and selectivity.

## 6 Sirtuin3 in Other Diseases

SIRT3 pathways are involved in pathophysiological processes associated with metabolic disorders. In addition to cancer, SIRT3 also plays important functions in multiple age-associated diseases, including hearing loss, obesity, diabetes, insulin resistance, neurodegeneration, cardiac hypertrophy, liver steatosis, and glucose homeostasis disorders (Giblin et al., 2014).

Since mitochondrial dysfunction is the basis of the pathogenesis of most neurodegenerative diseases, it is not surprising that the mitochondrial deacetylase SIRT3 plays a key role in several brain diseases, such as Parkinson’s disease (PD), Alzheimer’s disease (AD), Huntington’s disease (HD), and stroke (Gomes et al., 2020). Cheng et al. reported that SIRT3 serves as a vital molecule mediating neuroprotective adaptive stress responses. In HD and epilepsy models, hippocampal neurons and striatal vulnerability are increased in SIRT3^−/−^ mice. In the mouse temporal lobe epilepsy model, deficiency of or reduction in SIRT3 leads to hyperacetylation of some mitochondrial proteins, including SOD2 and cyclophilin D (Cheng et al., 2016). Thus, SIRT3 is required for neuroprotection because it improves mitochondrial function.

Cardiovascular diseases (CVDs) are the leading cause of death globally. Mitochondrial dysfunction plays a crucial role in CVD pathogenesis (Chistiakov et al., 2018). SIRT3 is a major and crucial mitochondrial NAD^+^-dependent deacetylase, that regulates most mitochondrial lysine acetylations. SIRT3 involved in cardiovascular physiology and pathology (Bugger et al., 2016). It has been reported that enhanced expression of SIRT3 protects myocytes from genotoxic and oxidative stress-mediated cell death, in part by blocking the transfer of Bax to mitochondria, as a stress-responsive deacetylase (Sundaresan et al., 2008). SIRT3 regulates the interaction of BaX and Ku70 by deacetylating Ku70. Sundaresan et al. proved that SIRT3 can reduce ROS levels and block cardiac hypertrophy. It was found that SIRT3 activates SOD2 and catalase to block cardiac hypertrophy in primary cardiomyocytes cultures, thereby decreasing ROS levels (Sundaresan et al., 2009). The hearts of SIRT3 knockout mice show accelerated signs of aging, manifested by cardiac hypertrophy and fibrosis. Thus, SIRT3 plays an indispensable role in preventing mitochondrial dysfunction and cardiac hypertrophy during aging (Hafner et al., 2010). In general, SIRT3 largely plays a protective role in myocardia.

Liver diseases have high morbidity, and there are more than 800 million cases (Marcellin and Kutala, 2018). SIRT3 levels in the liver are increased by fasting (Hirschey et al., 2010) and decreased by a high-fat diet (HFD) (Bao et al., 2010; Kendrick et al., 2011). In SIRT3 knockout mice, HFD feeding increases the acetylation of several hepatic proteins. This finding proves that SIRT3 is a key factor regulating hepatic mitochondrial function (Kendrick et al., 2011). Bao et al. found that palmitate enhances ROS and increases hepatocyte cell death in the absence of SIRT3. Restoring the level of SIRT3 and/or treatment with N-acetylcysteine, can ameliorate these adverse effects. SIRT3 ameliorates hepatic lipotoxicity, but paradoxically, downregulation of this adaptive protection occurs upon exposure to high fat in the liver (Bao et al., 2010).

SIRT3 can regulate a variety of mitochondrial functions to regulate fuel utilization. SIRT3 germline knockout mice develop various forms of metabolic dysfunctions at an accelerated rate, including obesity, hepatic steatosis, and insulin resistance (Hirschey et al., 2011). Zhang et al. found that SIRT3 is a positive regulator of macroautophagy and chaperone-mediated autophagy in adipocytes that promotes lipid mobilization *via* activating AMPK. SIRT3 might be a promising target for the treatment of obesity and related metabolic dysfunction (Zhang et al., 2020). It is crucial to preserve mitochondrial health to prevent insulin resistance and type 2 diabetes mellitus (T2DM) during aging. A major and early characteristic of the pathogenesis of T2DM is insulin resistance in skeletal muscle (DeFronzo and Tripathy, 2009). Jing et al. reported that mitochondrial dysfunction, increased oxidative stress, and JNK activation can be caused by decreased expression of SIRT3, which in turn induces impaired insulin signaling (Jing et al., 2011). The key factors mediating pancreatic *β* cell impairment in T2DM are chronic inflammation and mitochondrial dysfunction. SIRT3 mediates ROS production and plays a part in anti-inflammatory effects.

## 7 Conclusion

SIRT3 is an NAD^+^ -dependent deacetylase mainly located in mitochondria, that can regulate the activity of numerous substrate proteins to affect the fate of mitochondria, the nucleus, and even the entire cell. SIRT3 regulates oxidative stress, amino acid metabolism, fatty acid oxidation, electron transport, and the TCA cycle through deacetylating various substrate proteins (Kumar and Lombard, 2015). SIRT3 can coordinate complete shifts in mitochondrial metabolism and has a potential impact on diseases, especially CVDs and cancer (Chen et al., 2014). Studies using different cancer models to determine the precise role of SIRT3 in tumorigenesis have drawn different conclusions. SIRT3 plays a dual role in cancer, and whether it promotes or suppresses tumors probably depends on the type of cancer and the status of intracellular signaling pathways (Xiong et al., 2016). It appears that SIRT3 may prevent cell death by inhibiting oxidative stress; however, other studies have reported its function in promoting apoptosis (Torrens-Mas et al., 2017). By studying the mechanistic differences in various cancer types, our understanding of the carcinogenic and anticancer effects of SIRT3 may deepen, which can aid the development of novel cancer therapeutic strategies. Further characterization of the mitochondrial substrate protein of SIRT3 will contribute to a deeper understanding of cancer tumorigenesis. Such research may also help develop novel therapeutic strategies and improve patient outcomes. The identification of drugs that alter SIRT3 activity has become an important task. Despite the publication of a few relevant reports, no SIRT3 activator or inhibitor with excellent specificity and selectivity is currently being tested in a clinical trial. The activation pattern of SIRT3 has not been fully elucidated, which limits the development of SIRT3 inhibitors and activators. Therefore, monotherapies and combination therapies targeting SIRT3 hold substantial promise for drug development.

## Glossary

SIRT3: sirtuin 3
SIRTs: sirtuins
MTS: Mitochondrial Targeting Sequence
TCA: tricarboxylic acid
ROS: reactive oxygen species
IDH: isocitrate dehydrogenase
MnSOD: manganese superoxide dismutase
FOXO3a: forkhead transcription factor
PDC: pyruvate dehydrogenase complex
LCAD,long-chain acyl-CoA dehydrogenase
AceCS2: acetyl-CoA synthetase 2
HMGCS2: 3-hydroxy-3-methylglytaryl-CoA synthetase
GDH: Glutamate dehydrogenase
OTC: ornithine transcarbamylase
SDH: succinate dehydrogenase
mPTP: mitochondrial permeability transition pore
OGG1: 8-oxoguanine DNA glycosylase
SNPs: single nucleotide polymorphisms
VNTR: variable number of tandem repeats
BLCA: Bladder urothelial carcinoma
BRCA: Breast invasive carcinoma
CESC: Cervical squamous cell carcinoma
CHOL: Cholangiocarcinoma
COAD: Colon adenocarcinoma
ESCA: Esophageal carcinoma
GBM: Glioblastoma multiforme
HNSC: Head and Neck squamous cell carcinoma
KICH: Kidney Chromophobe
KIRC: Kidney renal clear cell carcinoma
KIRP: Kidney renal papillary cell carcinoma
LIHC: Live hepatocellular carcinoma
LUAD: lung adenocarcinoma
LUSC: lung squamous cell carcinoma
PAAD: Pancreatic adenocarcinoma
PRAD: Prostate adenocarcinoma
PCPG: Pheochromocytoma and paraganglioma
READ: Rectum adenocarcinoma
SARC: Sarcoma
SKCM: Skin Cutaneous melanoma
THCA: Thyroid carcinoma
THYM: Thymoma
STAD: Stomach adenocarcinoma
UCEC: Uterine corpus endometrial carcinoma
Tam: tamoxifen
ER: estrogen receptor
PYCR1: pyrroline-5-carpoxylate reductase-1
PTEN: Phosphatase and tensin homolog
NSCLC: non-small-cell lung cancer
NMNAT: nicotinamide mononucleotide adenylyltransferase
SCLC: small-cell lung cancer
HCC: Hepatocellular carcinoma
LDHA: lactate dehydrogenase A
SHMT2: Serine hydroxymethyltransferase 2
MPC1: Mitochondrial pyruvate carrier 1
RIPK3: Receptor-interacting serine/threonine-protein kinase 3
EMT: epithelial-mesenchymal transition
BAG-2: BCL2-associated athanogene protein
DLBCLs: Diffuse large B cell lymphomas
OSCC: Oral squamous cell carcinoma
JNK: Jun N-terminal kinase
ACC1: Acetyl CoA carboxylase 1
OA: Oroxylin A
PQQ: Pyrroloquinoline quinone
ITC: Isothermal Titration Calorimetry
MI/R: myocardial ischemia-reperfusion
α7nAChR: Alpha 7 nicotinic acetylcholine receptor
LVEF: left ventricular ejection fraction
LVFS: left ventricular fractional shortening
MM-GBSA: molecular mechanics/generalized Born surface area
PDHA1: pyruvate dehydrogenase E1α subunit
2-ME: 2-methoxyestradiol
AMC: 7-Amino-4-methylcoumarin
PD: Parkinson’s disease
AD: Alzheimer’s disease
HD: Huntington’s disease
CVDs: Cardiovascular diseases
HFD: high-fat diet
T2DM: type 2 diabetes mellitus
